# The risk factors for calcification vary among the different sections of the lower extremity artery in patients with symptomatic peripheral arterial disease

**DOI:** 10.1186/s12872-020-01615-w

**Published:** 2020-07-11

**Authors:** Hankun Yan, Zhihui Chang, Zhaoyu Liu

**Affiliations:** Department of Radiology, Shengjing Hospital of China Medical University, NO. 36, Sanhao Street, Heping District, Shenyang City, 110004 Liaoning Province China

**Keywords:** Arterial calcification, Peripheral arterial disease, Computed tomography, Lower extremity arterial calcification score, Lower extremity arterial stenosis index

## Abstract

**Background:**

Peripheral arterial disease (PAD) is associated with considerable mortality and morbidity worldwide. The present study explored the risk factors for arterial calcification among the different sections of the lower extremity in patients with PAD and analyzed their correlations with the extent of arterial stenosis at the corresponding section.

**Methods:**

This study enrolled symptomatic PAD patients from our hospital from March 2017 to March 2018. The lower extremity arterial calcification score (LEACS) and lower extremity arterial stenosis index (LEASI), representing the extent of arterial stenosis, were measured on computed tomography (CT) and the correlations between them were analyzed using Spearman’s correlation analysis. The relationships between risk factors and calcification were analyzed among the different sections of the lower extremity artery.

**Results:**

In total, 209 patients were included. The LEACSs of the total lower extremity, aortoiliac artery, and femoropopliteal and infrapopliteal arteries were correlated with the LEASI (all *P* < 0.05), but their correlation was relatively weak in the aortoiliac artery. Univariate analysis showed that hypertension was associated with the total (*P* = 0.019) and aortoiliac (*P* = 0.012) LEACSs. Diabetes was related to both femoropopliteal (*P* = 0.001) and infrapopliteal (*P* = 0.002) LEACSs. The infrapopliteal LEACS was higher in male patients (*P* = 0.011). After adjustment for age, the above relationships were maintained among the different sections, but not in the total lower extremity artery.

**Conclusions:**

The LEACS is associated with the LEASI in all arterial sections, but that of the aortoiliac artery was relatively weak. Different factors have different effects on calcification among the various sections of the lower extremity artery.

## Background

With improvements in living standards, changes in dietary habits, and prolongation of average lifespans, the morbidity rate of peripheral arterial disease (PAD) is increasing every year, which not only gravely affects people’s quality of life, but also increases their risk of death and limb ischemic events [1–3]. As a characteristic sign of PAD, arterial calcification has also been linked to adverse limb events and poor prognosis of interventional treatment and is associated with increased all-cause mortality [4–6]. Arterial calcification was previously considered a passive degenerative process but is currently recognized to be a complex process actively regulated by various cell molecules [7]. The abnormal deposition of extra osseous calcium salt in the human body occurs most frequently in the arterial system and causes arterial calcification [8, 9], mainly involving intima calcification and media calcification. However, these two types of arterial calcification cannot be accurately distinguished by conventional computed tomography (CT) [10]. According to the literature, arterial calcification is associated with traditional cardiovascular risk factors, such as diabetes, hypertension, hyperlipidemia, chronic kidney disease (CKD), and aging [11, 12]. Nonetheless, further studies have shown different mechanisms and prognoses of calcification in different vascular beds. Previous work has shown that arterial calcification is related to the apoptosis of vascular smooth muscle cells and macrophages and the release of matrix vesicles [13, 14]. In addition, some studies also showed that different types of arterial calcification (intima versus media) develop through various molecular mechanisms in different vessel types (large elastic versus smaller muscular arteries) and parts (proximal versus distal) [15, 16]. Allison et al. found that calcification of the thoracic, carotid, and iliac arteries was associated with overall mortality, whereas that of the coronary arteries was associated with cardiovascular disease mortality [17]. However, few studies have focused on the risk factors for calcification in the different sections of the lower limbs.

The relationship between calcification and arterial stenosis in the carotid and coronary vascular bed is well established [18, 19]. In contrast to calcification in carotid and coronary arteries, arterial calcification of PAD is most prominently located in the media and might even be more pronounced [20]. Therefore, the contribution of lower extremity arterial calcification (LEAC) to arterial stenosis may be different from in the neck and heart, and further exploration is necessary for the relationship between LEAC and the extent of arterial stenosis in patients with PAD. Quantification of LEAC has become readily available through semiautomated protocols using CT scans [21], and it has been widely used to predict mortality and assess the potential for successful interventional therapy [22–24]. In this study, a CT-based calcification score (CS) was used to evaluate the impact of cardiovascular risk factors on calcification in different sections of the lower extremity artery and to determine the correlations between the CS and the extent of arterial stenosis in different sections in patients with symptomatic PAD.

## Materials and methods

This retrospective, observational, single-center study included consecutive patients with symptomatic PAD who underwent CT angiography (CTA) in our hospital between March 2017 and March 2018. Approval for the study was obtained from our Institutional Review Board (IRB approval number: 2018PS225K), and patient consent was waived because of the retrospective nature of the study. We did not include patients presenting with acute embolism or in-stent restenosis or those with a prior amputation above the ankle. Baseline patient characteristics and individual risk factors collected included hypertension, hyperlipidemia, diabetes mellitus, CKD, and history of smoking and drinking.

### CT examination

In this study, CTA was performed with the following scanners: Brilliance iCT 256-slice CT scanner (Philips Healthcare, Cleveland, OH, USA), Ingenuity Core 128-slice CT scanner (Philips Healthcare, Cleveland, OH, USA), and Aquilion ONE 640-slice CT scanner (Toshiba Medical Systems, Otawara, Japan). The scan ranged from the abdominal aorta to the plantar artery. Briefly, 100 ml contrast medium (Iohexol, Shuangbei 350; Beilu Pharmaceutical Co., Ltd., Beijing, China) was injected into the middle vein of the right elbow at 4 ml/s via a 20-gauge intravenous cannula and a dual-cylinder high-pressure syringe (Ulrich REF XD 2051; Ulrich Medical GmbH, Ulm, Germany). Then, 40 ml normal saline was infused at the same rate.

### Measurement of the calcification score

The CS was measured from 3 cm of the abdominal aorta terminal to the ankle joint in plain images. The lower extremity artery was divided into three sections—aortoiliac arterial, femoropopliteal arterial, and infrapopliteal arterial—and each section included left and right corresponding arteries of the lower extremities. The aortoiliac arterial section included the abdominal aorta terminal, bilateral common iliac artery, and bilateral external iliac artery; the femoropopliteal arterial section included the bilateral superficial femoral artery and bilateral popliteal artery; and the infrapopliteal arterial section included the bilateral pretibial artery, bilateral tibiofibular diaphysis, bilateral post-tibial artery, and bilateral fibular artery. LEAC was measured on noncontrast CT scans. Calcification regions with a calcification area > 1 mm^2^ and density > 130 Hu were identified automatically using the open-source DICOM viewer (v4, OsiriX Imaging Software, Pixeo SARL) of Apple Macintosh (Apple Inc., Cupertino, CA, US). Bone tissues around target arteries from the abdominal aorta to the ankle joint were avoided; the target arterial sections were selected along the abdominal aorta (terminal 3 cm), iliac artery, femoropopliteal artery, and infrapopliteal artery. Finally, an overall CS was determined for each patient as the sum of all segmental CSs for both legs. Because the scores were skewed, log-transformed (CS + 1) values were used for all statistical analyses. Thus, we defined the log_10_ (CS + 1) values as the lower extremity arterial calcification score (LEACS). Two investigators who were blinded to the clinical status of the patients performed all calcium scoring after a brief training session. Evaluation of the inter- and intraobserver variabilities in the determination of the LEACS revealed Spearman correlation coefficients of 0.987 (*P* < 0.001) and 0.962 (*P* < 0.001), respectively [25].

### Measurement of the extent of arterial stenosis

In the contrast CT scans of the lower extremity CTA examination, the stenotic arterial sections were assessed independently by two radiologists. On the base of the three above sections, the lower extremity artery of each patient was further divided into 29 segments [26]. The aortoiliac arterial section included the abdominal aorta terminal, bilateral common iliac artery, and bilateral external iliac artery; the femoropopliteal arterial section included the bilateral deep femoral artery, bilateral superficial femoral artery (proximal and distal segments), and bilateral popliteal artery (proximal and distal segments); and the infrapopliteal arterial section included the bilateral pretibial artery (proximal and distal segments), bilateral tibiofibular diaphysis, bilateral post-tibial artery (proximal and distal segments), and bilateral fibular artery (proximal and distal segments). According to the lumen stenosis degree, the arterial stenosis degree was classified into nonsignificant stenosis (lumen diameter decreased by < 50%) and significant stenosis (lumen diameter decreased by ≥50%). The lower extremity arterial stenosis index (LEASI) was defined as the total number of arterial segments of the lower extremity with significant stenosis.

### Statistical analysis

Measurement data conforming to the normal distribution are expressed as mean ± standard deviation, non-normally distributed data are expressed as median (interquartile range), and count data are expressed as frequency and composition ratio. The correlations of the LEACS and the LEASI in the whole lower extremity and at each section were analyzed using Spearman’s correlation coefficient. By taking the median LEACSs (total, aortoiliac, femoropopliteal, and infrapopliteal arteries) as cutoff values, the patients were allocated into high and low calcification groups. Intergroup comparisons were performed using a chi-square test. The risk factors for arterial calcification in the total lower extremity and at each section were assessed by univariate linear regression analysis. Multivariate linear regression analysis was used to determine the independent risk factors associated with the LEACS and obtain the partial regression coefficient (PRC), 95% confidence interval (CI), and *P* value. All statistical analyses were performed with SPSS 25.0 software and all statistical tests were two-tailed. *P* < 0.05 indicated that the difference was statistically significant.

## Results

**Fig. 1:**
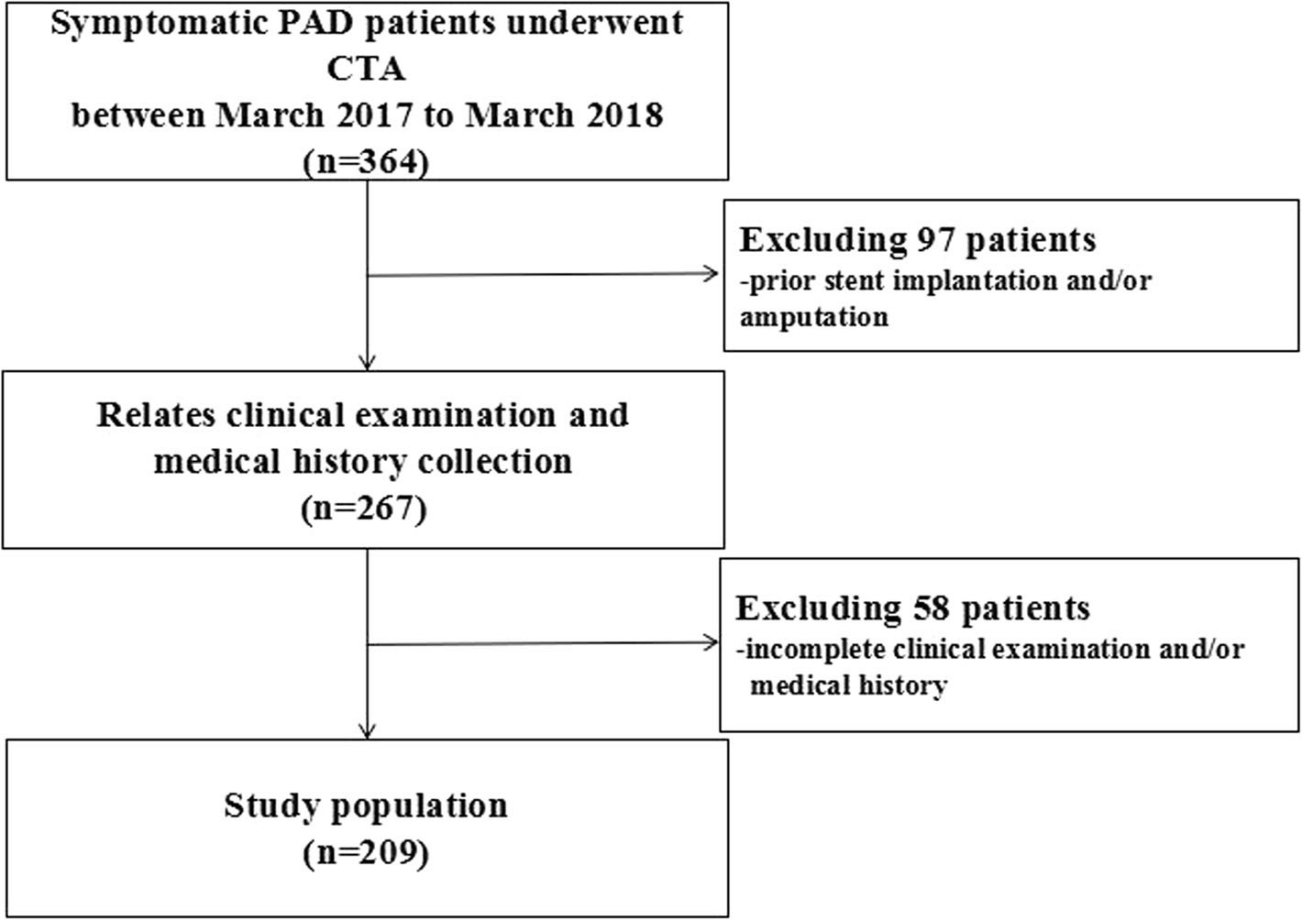
Patient selection flow chart

A total of 209 patients were included in this study. The patient selection process is detailed in Fig. 1. Patients’ basic characteristics are shown in Table 1.

**Table 1:** Basic characteristics of the patients

Characteristics	PAD patients
Number of patients (n)	209
Age (year)	66.3 ± 10.2
Age > 60 years [n (%)]	149 (71.3)
Smoking [n (%)]	148 (70.8)
Drinking [n (%)]	92 (44.0)
Male [n (%)]	164 (78.5)
Diabetes [n (%)]	112 (55.3)
Hyperlipidemia [n (%)]	93 (53.6)
Hypertension [n (%)]	133 (63.6)
CKD [n (%)]	49 (23.4)
Total LEACS	3.7 ± 0.8
Aortoiliac LEACS	3.5 ± 0.7
Femoropopliteal LEACS	3.0 ± 1.2
Infrapopliteal LEACS	2.5 ± 1.6
Total LEASI	12.6 ± 7.2
AortoiliacLEASI	1.1 ± 1.4
Femoropopliteal LEASI	4.9 ± 3.2
Infrapopliteal LEASI	6.7 ± 5.0

Note: *CKD* chronic kidney disease, *LEACS* lower extremity arterial calcification score, *LEASI* lower extremity arterial stenosis index

**Fig. 2:**
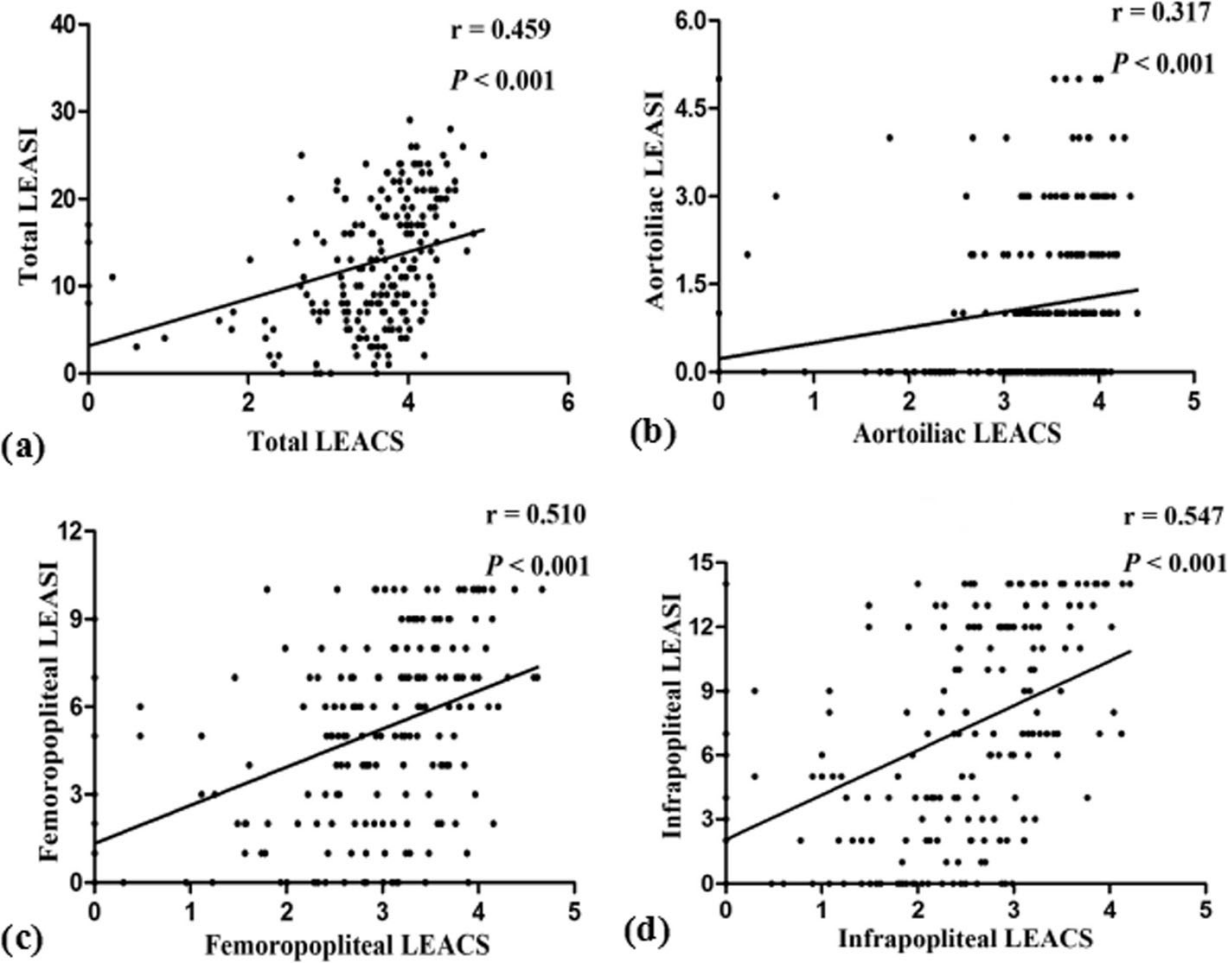
Correlation between the LEACS and the LEASI. **a** Scatter diagram showing a positive correlation between the total LEACS and the total LEASI (*r* = 0.459, *P* < 0.001). **b** Scatter diagram showing a positive correlation between the aortoiliac LEACS and the aortoiliac LEASI (*r* = 0.317, *P* < 0.001). **c** Scatter diagram showing a positive correlation between the femoropopliteal LEACS and the femoropopliteal LEASI (*r* = 0.510, *P* < 0.001). **d** Scatter diagram showing a positive correlation between the infrapopliteal LEACS and the infrapopliteal LEASI (*r* = 0.547, *P* < 0.001). LEACS, lower extremity arterial calcification score; LEASI, lower extremity arterial stenosis index

According to the lower extremity CTA examination, 627 sections of the lower extremity artery, encompassing a total of 6061 segments, were assessed. Significant stenosis was found in 2639 segments (43.5%). The total LEACS was associated with the total LEASI (*r* = 0.459, *P* < 0.001). As for each arterial section, the LEACSs of the aortoiliac artery (*r* = 0.317, *P* < 0.001), femoropopliteal artery (*r* = 0.510, *P* < 0.001), and infrapopliteal artery (*r* = 0.547, *P* < 0.001) were all correlated with the LEASI, but their correlation was relatively weak in the aortoiliac artery (Fig. 2).

The correlations among the various arterial sections of the lower extremity (including the aortoiliac artery, femoropopliteal artery, and infrapopliteal artery) and the total LEACS are shown in Table 2. The LEACS at each arterial section of the lower extremity and the LEACS at the left/right lower extremity artery were strongly associated with the total LEACS. A high correlation was also found in the LEACS between the left and right lower extremities. Among the three sections of the lower extremity, the highest correlation with total LEACS was observed at the femoropopliteal arterial section.

**Table 2:** Correlation analysis of the LEACS among various sections

	r	*P*
Total LEACS vs. Aortoiliac LEACS	0.890	< 0.001
Total LEACS vs. Femoropopliteal LEACS	0.893	< 0.001
Total LEACS vs. Infrapopliteal LEACS	0.796	< 0.001
Total LEACS vs. Left LEACS	0.976	< 0.001
Total LEACS vs. Right LEACS	0.974	< 0.001
Left LEACS vs. Right LEACS	0.931	< 0.001

Note: *LEACS* lower extremity artery calcification score

With the median LEACSs of the total lower extremity and each arterial section as cutoffs, the patients were further allocated into high and low CS groups (Table 3). As shown by analysis of the total lower extremity and each arterial section, the LEACSs of patients aged > 60 years were higher in the total lower extremity and all arterial sections. Furthermore, the LEACSs in the total lower extremity and aortoiliac arterial section were higher in patients with hypertension, whereas those in the femoropopliteal and infrapopliteal arterial sections were higher in patients with diabetes. Interestingly, there were more male patients in the high CS group of the infrapopliteal arterial section.

**Table 3:** Comparison of baseline data between the high and low CS groups of the whole lower extremity and various arterial sections

	Total lower extremity			Aortoiliac artery			Femoropopliteal artery			Infrapopliteal artery		
	High CS (*n* = 104)	Low CS (*n* = 105)	*p*	High CS (*n* = 104)	Low CS (*n* = 105)	*p*	High CS (*n* = 104)	Low CS (*n* = 105)	*p*	High CS (*n* = 104)	Low CS (*n* = 105)	*p*
Age > 60 years	84 (80.8)	65 (61.9)	0.003	84 (80.8)	65 (61.9)	0.003	85 (81.7)	64 (61.0)	0.001	83 (79.8)	66 (62.9)	0.007
Male	85 (81.7)	79 (75.2)	0.254	86 (82.7)	78 (74.3)	0.139	82 (78.8)	82 (78.1)	0.837	90 (86.5)	74 (70.5)	0.011
Smoking	76 (73.1)	72 (68.6)	0.474	80 (76.9)	68 (64.8)	0.053	70 (67.3)	78 (74.3)	0.267	73 (70.2)	75 (71.4)	0.844
Drinking	49 (47.1)	43 (41.0)	0.370	49 (47.1)	43 (41.0)	0.370	48 (46.2)	44 (41.9)	0.536	49 (47.1)	43 (41.0)	0.370
Diabetes	61 (58.7)	51 (48.6)	0.144	56 (53.8)	56 (53.3)	0.941	63 (60.6)	49 (46.7)	0.044	67 (64.4)	45 (42.9)	0.002
Hypertension	75 (72.1)	58 (55.2)	0.011	74 (71.2)	59 (56.2)	0.025	71 (68.3)	62 (59.0)	0.166	67 (64.4)	66 (62.9)	0.814
Hyperlipidemia	46 (44.2)	47 (44.8)	0.938	45 (43.3)	48 (45.7)	0.722	43 (41.3)	50 (47.6)	0.362	44 (42.3)	49 (46.7)	0.526
CKD	25 (24.0)	24 (22.9)	0.840	23 (22.1)	26 (24.8)	0.652	28 (26.9)	21 (20.0)	0.238	25 (24.0)	24 (22.9)	0.840

Note: *CS* calcification score, *CKD* chronic kidney disease

As shown by univariate linear regression analysis, the total LEACS was associated with age > 60 years, diabetes, and hypertension. Multivariate analysis indicated that only age > 60 years was an independent factor influencing the total LEACS. To determine the risk factors for different arterial sections, univariate and multivariate linear regression analyses were performed for each section of the lower extremity. After adjustment for age, the results showed that hypertension was an independent influencing factor for the aortoiliac LEACS, whereas diabetes was an independent influencing factor for the femoropopliteal and infrapopliteal LEACSs. In addition, male sex was an independent risk factor for the infrapopliteal LEACS (Tables 4 and 5).

**Table 4:** Univariate linear regression analysis of the LEACS in the total lower extremity artery and at various arterial sections

Factors	Total lower extremity artery		Aortoiliac artery		Femoropopliteal artery		Infrapopliteal artery	
	PRC (95%CI)	*P*	PRC (95%CI)	*P*	PRC (95%CI)	*P*	PRC (95%CI)	*P*
Age > 60 years	0.744 (0.496–0.992)	< 0.001	0.650 (0.397–0.903)	< 0.001	0.988 (0.650–1.325)	< 0.001	0.840 (0.503–1.177)	< 0.001
Male	0.50(−0.043–0.543)	0.094	0.184(−0.110–0.478)	0.219	0.199(− 0.201–0.598)	0.328	0.504 (0.119–0.890)	0.011
Diabetes	0.263 (0.023–0.504)	0.032	0.098(−0.145–0.341)	0.429	0.564 (0.244–0.885)	0.001	0.504 (0.189–0.820)	0.002
Smoking	0.100(−0.167–0.366)	0.461	0.238(−0.027–0.503)	0.078	− 0.004(− 0.366–0.358)	0.983	−0.051 (0.405–0.303)	0.778
Drinking	0.090(−0.154–0.334)	0.469	0.099(−0.145–0.343)	0.425	0.152(− 0.179–0.482)	0.367	0.312(− 0.009–0.634)	0.057
CKD	0.099(− 0.187–0.385)	0.496	−0.116(− 0.402–0.170)	0.424	0.310(− 0.076–0.696)	0.114	0.369(− 0.008–0.746)	0.055
Hypertension	0.297 (0.048–0.546)	0.019	0.320 (0.072–0.568)	0.012	0.269(− 0.071–0.609)	0.120	0.170(− 0.164–0.504)	0.317
Hyperlipidemia	0.0.93(− 0.151–0.337)	0.452	0.121(− 0.122–0.365)	0.327	0.170(− 0.161–0.500)	0.313	−0.043(− 0.367–0.281)	0.795

Note: *CKD* chronic kidney disease

**Table 5:** Multivariate linear regression analysis of the LEACS in the total lower extremity artery and at various arterial sections

Factors	Total lower extremity artery		Aortoiliac artery		Femoropopliteal artery		Infrapopliteal artery	
	PRC (95%CI)	*P*	PRC (95%CI)	*P*	PRC (95%CI)	*P*	PRC (95%CI)	*P*
Age > 60 years	0.703 (0.456–0.951)	< 0.001	0.616 (0.362–0.869)	< 0.001	0.950 (0.620–1.281)	< 0.001	0.889 (0.560–1.219)	< 0.001
Male	–	–	–	–	–	–	0.556 (0.188–0.924)	0.003
Diabetes	0.184(−0.043–0.411)	0.112	–	–	0.507 (0.207–0.806)	0.001	0.450 (0.155–0.745)	0.003
Hypertension	0.194(−0.040–0.429)	0.104	0.242 (0.006–0.477)	0.045	–	–	–	–

## Discussion

In this study, the total LEACS was associated with the total LEASI, indicating that the extent of arterial stenosis could be better predicted by the degree of calcification. This finding is supported by several similar observations [23, 27]. The internal diameter of blood vessels in the normal human body can be maintained through a compensatory increase in arterial perimeter, but Sigrist et al. [28] found that decreased arterial compliance is associated with femoral arterial calcification in dialysis patients, which might eventually lead to vascular stenosis. Similar results have been obtained in several studies [29, 30]. Furthermore, we found that the LEACS was associated with the LEASI at the aortoiliac, femoropopliteal, and infrapopliteal arterial sections but showed a relatively weak correlation in the aortoiliac artery. This may be due to the different anatomical structures and hemodynamics of the lower extremity arteries, meaning that the influence of the calcification of different sections on the degree of arterial stenosis might differ. Because the diameter of the iliac artery is larger, it is less affected by the calcification of the vascular wall. In contrast, the distal femoropopliteal artery and infrapopliteal artery gradually become thinner, resulting in a greater influence of calcification.

Cardiovascular disease risk factors partially mediate the association between incident mortality and calcification in the different vascular beds [17]. The presence of calcification in the carotid or coronary arteries is indicative of a higher atherosclerotic burden and is associated with lower extremity arterial calcification in patients with PAD [17, 26]. It would thus be valuable to describe the extent of the carotid or coronary arteral calcification. However, because only a few of the patients included in this study underwent carotid or coronary CT examination at the same time, only the lower extremity artery was involved in this study. The LEACS increased with age in patients with PAD in this study. Diabetes is associated with increased arterial calcification [31]. Hyperlipidemia is also a common risk factor for arterial calcification, especially in the coronary artery. In addition, some studies demonstrated that lipid-lowering agents could delay the calcification process [32]. Vascular calcification is more common and severe and develops earlier in patients with CKD or on dialysis [33]. However, the total LEACS was not significantly correlated with diabetes, CKD, or hypertension in this study, possibly because the sample size was insufficient or there was a sampling error.

The LEACS was also analyzed at each arterial section of the lower extremity in our study. In the high CS groups of the femoropopliteal and infrapopliteal arterial sections, the proportions of patients > 60 years old and with diabetes were higher; meanwhile, in the high CS group of the aortoiliac arterial section, the proportion of patients > 60 years old and with hypertension was higher. As shown by multivariate linear regression analysis, after adjustment for age, the independent influencing factors for each arterial section of the lower extremity were different: hypertension and diabetes were independent risk factors for the aortoiliac LEACS and the femoropopliteal and infrapopliteal LEACSs, respectively, suggesting that the mechanisms for calcification at different arterial sections of the lower extremity might not be entirely the same and that the distribution of the LEACS is influenced by different risk factors. Previous work showed that compensatory arterial dilation in diabetic patients was restrained to exacerbate distal arteriosclerosis during the progression process of PAD [34]. Therefore, distal arterial calcification of the lower extremity is more serious in diabetic patients. In PAD patients with hypertension, the hemodynamic change plays a dominant role in the occurrence of lesions, so the proximal arteries of the lower extremity are more greatly influenced and may thus be more severely calcified, which correlates with our findings. In addition, male sex was also an independent risk factor for a higher infrapopliteal LEACS in this study, and the proportion of male sex was higher in the high CS group of the infrapopliteal arterial section, which might be related to estrogen. As an important physiological hormone for female development, estrogen can increase bone density by inhibiting the activity of osteoclasts and thus also plays an important role in the metabolism of bone tissue. Previous studies have shown that serum estradiol levels are negatively correlated with coronary arterial calcification and that arterial calcification can be reduced by estrogen therapy [35, 36]. Other work revealed that patient prognosis was influenced by arterial calcification in different sections of the lower extremity [37, 38]. Therefore, clinicians might preliminarily judge the distribution and prognosis of LEAC by identifying patients’ relevant risk factors. In addition, the calcification of different sections might reflect the existence of different disease states. Furthermore, although arterial calcification is related to the degree of arterial stenosis, there is no clinically targeted drug that inhibits calcification, and its progress can thus only be indirectly slowed down through risk factor control. Our results suggest that, in future clinical practice, we will be able to control the related risk factors on a patient-specific basis according to the characteristics of calcification distribution in the lower extremity arteries.

Although the LEACS can effectively predict prognosis [5, 26], it is very time-consuming to measure the LEACS in the whole lower extremity artery in the clinical setting. In this study, the LEACS at each arterial section of the lower extremity and in both lower extremities were highly correlated with the total LEACS, especially in the femoropopliteal arterial sections. This suggests that it might be feasible to use the LEACS of a single arterial section, such as the femoropopliteal arterial section, to represent the total LEACS. Sook et al. [4] found that increased calcification of the tibial artery was associated with an increase in the failure rate of angioplasty and the incidence of nonscheduled amputation. Another study also suggested that calcification of the suprapatellar artery was associated with severe limb ischemia in patients on hemodialysis [4, 10]. However, these associations should be further investigated in a large-scale study, given that the studies were single-center studies with small sample size and number of adverse events.

There are some limitations to our work. This study was a retrospective, small-sample, single-center study, which might lead to bias. Another important limitation was that, due to the retrospective design, we were unable to obtain detailed information on whether patients were treated systematically for combined disease. This might be important because some studies have shown that metformin and statins can inhibit calcification [32], which might affect the results. Nonetheless, this is the first study to analyze calcification in different lower limb sections, which could help to better explain the pathophysiology of arterial calcification. Further work with a larger sample size is nonetheless needed.

## Conclusions

The LEACS is associated with the extent of arterial stenosis in all arterial sections, although the association was relatively weak in the aortoiliac artery. Traditional cardiovascular risk factors have varying effects on calcification among the different arterial sections of the lower extremity artery. Our results suggest that we should control the corresponding risk factors according to the distribution characteristics of LEAC to delay the calcification process.

## Data Availability

The datasets used and/or analyzed during the current study are available from the corresponding author on reasonable request.

## Abbreviations

PAD: Peripheral arterial disease
LEACS: Lower extremity arterial calcification score
LEASI: Lower extremity arterial stenosis index
CT: Computed tomography
CTA: Computed tomography angiography
CKD: Chronic kidney disease
LEAC: Lower extremity arterial calcification
CS: Calcification score
PRC: Partial regression coefficient
CI: Confidence interval
